# Hyperspectral imaging in the emergency department to characterize lower leg edema

**DOI:** 10.64898/2026.09.15.26363173

**Published:** 2026-09-16

**Authors:** Karthik Kasi, Cameron May, Hailey Gallegos, Leo Kobayashi, Bevil R. Conway, Joseph R. Pare

## Abstract

Interactions between light and biological tissue could reveal disease-related changes that could be advantageous for rapid diagnosis when applied to whole limbs. Here, we tested the extent to which visible/near-infra-red hyperspectral imaging (HSI) deployed in the emergency department could classify cellulitis, non-cellulitis edema, and healthy lower-leg tissue across skin pigmentation types. We collected HSI data of the lower leg from 83 emergency-room patients and analyzed the calibrated normalized spectra (400-1000 nm) using a machine-learning classification algorithm, evaluated by cross-validation and benchmarked against standard spectral indices (oxygenation, hemoglobin, water, near-infrared perfusion). Classification at patient-level and pixel-level resolution performed comparably well using whole-spectrum and indices, for cellulitis versus healthy (AUC: 0.93 vs. 0.96) and non-cellulitis edema versus cellulitis (AUC: 0.92 vs. 0.89). Full-spectrum analysis substantially improved non-cellulitis edema versus healthy classification (patient-level AUC: 0.72 vs. 0.41; pixel-level AUC: 0.78 vs. 0.50), with no significant effect of skin type on accuracy.

## Full Text Availability

The license terms selected by the author(s) for this preprint version do not permit archiving in PMC. The full text is available from the preprint server.

